# A Mean of Three-Year Follow-Up of Transvaginal Mesh Repair Using Calistar System Devices for the Treatment of Pelvic Organ Prolapse

**DOI:** 10.3390/jcm14134703

**Published:** 2025-07-03

**Authors:** Chao-Chi Huang, Kun-Ling Lin, I-Chieh Sung, Zixi Loo, Cheng-Yu Long

**Affiliations:** 1 Department of Obstetrics and Gynecology, Cheng Ching Hospital, Taichung 40764, Taiwan; dbdbdbdb3@hotmail.com; 2 Department of Obstetrics and Gynecology, Kaohsiung Medical University Hospital, Kaohsiung 80708, Taiwan; nancylin95@gmail.com (K.-L.L.); starrynight278@gmail.com (I.-C.S.); loozixi@hotmail.com (Z.L.)

**Keywords:** pelvic organ prolapse, transvaginal mesh, calistar system, urinary incontinence, urodynamics

## Abstract

**Background/Objectives:** Pelvic organ prolapse (POP) and urinary incontinence are prevalent conditions among women, significantly affecting their quality of life. Vaginal mesh surgeries, including the use of the Calistar mesh, have become an essential intervention aimed at alleviating symptoms associated with POP and urinary dysfunction. This study evaluates the clinical outcomes of Calistar vaginal mesh surgeries, focusing on pre- and post-operative changes in urinary parameters and prolapse severity. **Methods:** Data from 180 patients undergoing Calistar procedures were analyzed, revealing significant improvements in anatomical markers (Aa, Ba, C, Ap, and Bp) and urinary distress metrics (UDI-6 and IIQ-7) postoperatively. **Results:** The results demonstrate that Calistar mesh procedures are effective in reducing urinary frequency, incontinence, and incomplete bladder emptying. **Conclusions:** Calistar mesh procedures offer a safe and effective surgical option for managing POP and associated urinary dysfunction. The observed anatomical and functional improvements suggest that Calistar mesh significantly enhances patient outcomes and quality of life.

## 1. Introduction

Pelvic organ prolapse (POP) is a common and progressive condition among women, particularly affecting those with advanced age and a multiparous history. The pathophysiology involves weakening of the pelvic floor support structures, including muscles, ligaments, and connective tissue, leading to the descent of pelvic organs such as the bladder, uterus, or rectum into or through the vaginal canal. The global burden of POP is increasing with aging populations, and it is estimated that up to 50% of parous women exhibit some degree of prolapse upon clinical examination, although not all are symptomatic. Nevertheless, a significant subset experiences substantial impairment in their quality of life due to symptoms such as pelvic pressure, vaginal bulging, urinary incontinence, voiding dysfunction, and sexual difficulties [1,2].

Traditional approaches to managing POP range from conservative measures, including pelvic floor rehabilitation and pessary use, to surgical interventions. Among surgical strategies, native tissue repair has long been the mainstay; however, its high recurrence rate—reportedly up to 30% in some series—has led to the exploration of mesh-augmented procedures [3]. Transvaginal mesh (TVM) surgeries were introduced to reinforce weakened pelvic tissues, with the goal of improving long-term durability and anatomical outcomes. Over the past two decades, a variety of mesh kits have been developed, with the Calistar system being among the newer-generation devices designed to reduce complications associated with earlier models through improved mesh weight, porosity, and anchoring design [4,5].

Despite their initial promise, transvaginal mesh procedures have generated considerable controversy. Regulatory agencies such as the U.S. FDA and health authorities in Europe have issued warnings or even withdrawn certain mesh products from the market due to concerns over complications such as mesh erosion, pelvic pain, infection, and dyspareunia [6,7,8]. These issues have driven a global shift toward either mesh-free techniques or the development of safer mesh alternatives with improved design features. Nevertheless, the high recurrence rate of native tissue repairs and the persistent need for durable anatomical support have sustained clinical interest in the use of improved mesh systems [9,10].

In Taiwan, the use of vaginal mesh is still permitted under strict institutional review. At our center, the Calistar system has been approved for clinical use following internal evaluation of safety and design improvements compared to previous-generation mesh kits. All mesh-based repairs follow standardized protocols based on national urogynecological society guidelines, with informed consent obtained from each patient.

Furthermore, POP frequently coexists with lower urinary tract symptoms (LUTS), including urgency, frequency, stress incontinence, and incomplete bladder emptying [11]. Although surgical correction of POP often improves these symptoms, the correlation between anatomical success and functional recovery is not always linear. In some cases, postoperative LUTS persist or even emerge de novo. The role of urodynamic studies (UDS) in preoperative evaluation and postoperative follow-up remains debated. While Nager et al. (2012) questioned the utility of routine preoperative urodynamics [12], other research—including Long et al. (2011)—suggested that specific preoperative bladder characteristics, such as high bladder capacity or detrusor overactivity, may predict symptom improvement following TVM repair [11].

More recent studies, such as Naumann et al. (2021) and Long et al. (2024), have further highlighted the mid-term safety and functional benefits of Calistar mesh in anatomically complex or recurrent POP cases [4,5]. However, few have incorporated both urodynamic and patient-reported outcomes into a structured longitudinal follow-up.

In this study, we aim to bridge this knowledge gap by presenting a three-year follow-up of 180 patients who underwent Calistar mesh repair for symptomatic POP. Our objectives were to evaluate the degree of anatomical correction using the POP-Q system, assess subjective improvements through validated LUTS-related questionnaires (UDI-6 and IIQ-7), and analyze changes in objective urodynamic parameters. By integrating these outcome domains, we aim to contribute further evidence regarding the safety and efficacy of the Calistar system in contemporary POP management.

## 2. Materials and Methods

From January 2019 through December 2021, the medical records of women receiving single incision mesh surgery with Calistar system at our hospital were retrospectively reviewed. This study was designed as a single-center retrospective cohort analysis.

Inclusion criteria were

(1) Age ≥ 18 years;
(2) Stage II or greater anterior and/or apical pelvic organ prolapse (POP), as defined by the Pelvic Organ Prolapse Quantification (POP-Q) system,
(3) availability of complete preoperative and postoperative data with a minimum of 12 months of follow-up.

Exclusion criteria were:

(1) incomplete medical records,
(2) loss to follow-up within 12 months after surgery.

A total of 180 women met the inclusion criteria and were included in the final analysis. All patient data were anonymized. All surgeries were performed by a single experienced urogynecologic surgeon using a standardized operative protocol. Data extraction followed a standardized procedure, including operative notes, clinical visits, and imaging or urodynamic reports. The Calistar system was selected for patients with anterior and/or apical prolapse. The decision to proceed with mesh surgery was based on prolapse severity, failure of conservative treatment, patient preference, and informed consent. Patients undergoing mesh repair for recurrent prolapse or concomitant urinary incontinence were not excluded.

Although prior studies, such as Naumann et al. (2021), have reported favorable anatomical and functional outcomes using single-incision mesh systems like Elevate, most available data have focused primarily on anatomical results, often in small case series, and lacked objective bladder function evaluation [5]. There remains limited evidence assessing mid-term lower urinary tract symptoms (LUTS) and urodynamic performance following Calistar placement. This study was therefore designed to address this gap by evaluating both anatomical outcomes and functional changes in LUTS and bladder physiology after Calistar mesh implantation. In addition, this study was conducted as a single-center retrospective cohort, and all procedures were performed by a single experienced urogynecologic surgeon following a standardized institutional protocol.

The Calistar system was chosen over other transvaginal mesh devices due to its ultra-lightweight, macroporous design and dual anchoring approach, which have been associated with reduced mesh-related complications and improved functional outcomes in comparative studies [7,13,14]. Although the central portion of the mesh is extremely thin, this does not appear to compromise its success rate. Its single-incision delivery technique also allows for a less invasive approach with faster recovery. Device selection in our study was based on surgeon preference, patient anatomy, prolapse severity, and informed consent. All patients underwent consistent preoperative evaluation, mesh selection, and postoperative follow-up procedures, according to hospital protocol.

POP was assessed preoperatively and postoperatively using the Pelvic Organ Prolapse Quantification (POP-Q) system. Measurements including Aa, Ba, C, Ap, Bp, and total vaginal length (TVL) were recorded and evaluate changes in anatomical correction following surgery. Subjective symptoms and quality of life were evaluated using the Urinary Distress Inventory-6 (UDI-6) and Incontinence Impact Questionnaire-7 (IIQ-7) [15]. These validated questionnaires were completed by participants before and after surgery to assess LUTS and their impact on daily life. Each POP-Q examination was performed in the lithotomy position during maximal Valsalva maneuver to ensure reproducibility. The UDI-6 and IIQ-7 questionnaires were administered in person by a trained research assistant, and patients were encouraged to complete them independently in a private setting to reduce bias. For illiterate or visually impaired patients, assistance was provided verbally in a standardized, non-leading manner. The use of these questionnaires is standard practice in our institution, and both have been validated for use in the local language to ensure clarity and consistency in daily clinical application.

Urodynamic studies (UDS) were performed both preoperatively and postoperatively to objectively measure bladder function and voiding patterns. The parameters evaluated included maximum urinary flow rate (Qmax), residual urine volume (RU), first sensation of bladder filling (FS), maximum cystometric capacity (MCC), detrusor pressure at maximum flow (Pdet), maximal urethral closure pressure (MUCP), functional urethral length (FUL), and urethral closure area (UCA). Detrusor overactivity (DO) was identified during the filling phase of cystometry if uninhibited detrusor contractions were observed.

In addition to anatomical results, this study emphasized urodynamic testing as a key method to objectively evaluate the functional impact of Calistar mesh implantation—an aspect underrepresented in prior mesh-based prolapse repair studies.

### 2.1. Operative Technique: The Calistar System

The Calistar-S mesh, designed for targeted anterior and apical compartment reinforcement via single-incision transvaginal access, is introduced transvaginally through a single-incision approach to reinforce the anterior vaginal wall. It preserves the minimally invasive nature of traditional TVM while incorporating advancements in anatomical anchoring and biocompatible mesh design. Precise placement and fixation are critical to achieving optimal anatomical restoration while minimizing complications. The anterior attachment arms of the mesh are positioned bilaterally within the obturator internus muscles using the Retractable Insertion Guide (RIG). This ensures secure fixation and adequate anterior compartment support. The posterior arms are anchored to the sacrospinous ligaments utilizing the Tissue Anchoring System (TAS), which provides stable fixation without excessive tension. Recent studies have highlighted that mesh characteristics such as weight, pore size, and stiffness have a significant impact on patient outcomes. For example, Wang et al. (2021) emphasized that meshes with higher stiffness are associated with vaginal degeneration and functional impairment [13], a finding supported by biomechanical investigations from Liang et al. (2013) [15]. In response to these findings, lighter, macroporous, and anatomically anchored mesh systems—such as Calistar-S and Calistar-A—have been developed to reduce adverse effects while maintaining adequate prolapse correction. In a recent guideline by Deffieux et al. (2024), the Calistar system was noted among the contemporary options with lower complication rates and promising outcomes [7].

Following placement, the mesh is carefully adjusted to restore the normal pelvic anatomical relationship while avoiding overcorrection, which could predispose patients to postoperative voiding dysfunction. Adequate tensioning is confirmed intraoperatively, ensuring that the mesh provides support without creating undue rigidity or distortion of the vaginal axis. In cases with concomitant stress urinary incontinence (SUI), a mid-urethral sling was placed using either retropubic or transobturator approach. The decision was based on preoperative evaluation and patient preference. Concomitant hysterectomy or cervical amputation was also performed in patients with uterine prolapse, adenomyosis, or cervical elongation, using vaginal or laparoscopic assistance depending on pelvic anatomy and surgical complexity.

As a follow-up, postoperative outpatient visits were at 1, 2, 3, 6, and 12 months and then semiannually beyond one year. Pelvic examination was performed routinely in every visit to clinics. The primary outcome of the study was anatomical correction rate after the surgery, assessed objectively by pelvic examination using the POP-Q system. Secondary outcomes included assessment of subjective LUTS, objective UDS performance, intra- and postoperative complication, and continence rate. Recurrence was defined as POP-Q stage 2 or more found at any follow-up visit. Postoperative voiding dysfunction was defined as RU more than 100 mL under ultrasound after twice micturition. Recurrence was managed conservatively in asymptomatic patients or with repeat surgery in symptomatic cases. Mesh exposure, if present, was documented, photographed, and treated with estrogen therapy or surgical excision. Data on sexual function were not collected formally in this study but were discussed during follow-ups at patient discretion. The follow-up protocol, including physical exam, questionnaire completion, and urodynamic testing when indicated, was standardized for all participants across the study period. No protocol deviations were recorded.

### 2.2. Statistics

IBM SPSS Statistical Software version 20.0 ed. was used for statistical analyses. The paired *t*-test was performed for comparison between pre-operative and post-operative POP-Q parameters and two related units on a continuous outcome. The McNemar’s and Fisher’s exact tests were performed for categorical variables. A *p*-value of less than 0.05 was considered statistically significant.

### 2.3. Ethical Approval

This study received approval from the Institutional Review Board of Kaohsiung Medical University Hospital (ID: KMUHIRB-E(I)-20190015), by which relevant guidelines and regulations were followed accordingly. Limitations of the study include its retrospective design, lack of randomization, and absence of a control group. Although data collection and follow-up protocols were standardized, potential selection bias and residual confounding may still exist. These limitations should be considered when interpreting the study findings.

## 3. Results

A total of 180 women were enrolled in the study, with a mean age of 68.6 years (SD ± 8.2) (Table 1). The average parity was 3.16 (SD ± 1.26), and the mean body mass index (BMI) was 24.64 (SD ± 3.13). Among the participants, 42.78% (77 women) were postmenopausal at the time of surgery, and 40% (72/180) women had a history of hypertension. Diabetes mellitus was present in 20% (36/180) women, while 40% (72 women) had previous hysterectomy. Concomitant anti-incontinence surgery were performed in 85 women (85/180; 47.22%) following TVM procedures.

Postoperatively, notable enhancements were observed across multiple clinical parameters (Table 2). All parameters of Pelvic Organ Prolapse Quantification (POP-Q) system, including Point Aa, Ba, C, Ap, Bp, and TVL, demonstrated significant improvement. (*p* < 0.01). Success rate of 96.1% (173/180) was achieved after a follow-up of 12–72 months.

Regarding the validated questionnaires of quality of life, the mean Urinary Distress Inventory-6 (UDI-6) score decreased from 27.78 to 5.34 (*p* < 0.01), and the mean scores of Incontinence Impact Questionnaire-7 (IIQ-7) dropped from 31.09 to 2.78 (*p* < 0.01). Residual urine volume decreased significantly from 132.50 mL to 54.51 mL (*p* < 0.01).

The majority of lower urinary symptoms showed substantial improvement following surgery, except symptoms of urge incontinence and nocturia (Table 3). The prevalence of urinary frequency decreased from 57.69% to 1.28% (*p* < 0.01), while incomplete bladder emptying declined from 49.02% to 7.84% (*p* < 0.01) (Table 3). Urinary hesitancy was reduced from 69.62% to 5.06% (*p* < 0.01).

As for urodynamic parameters, only residual urine exhibited a statistically significant decrease (*p* < 0.01) (Table 4). Other parameters, including maximum urinary flow rate, maximum cystometric capacity, functional urethral length, and maximum urethral closure pressure, did not show significant changes postoperatively (*p* > 0.05).

## 4. Discussion

Based on a comprehensive review of the current literature, the following discussion integrates findings from multiple studies to contextualize and interpret the results of our investigation into surgical interventions for POP and associated LUTS.

### 4.1. Pelvic Organ Prolapse and Surgical Outcomes

POP is a prevalent condition among women, particularly in postmenopausal populations. Surgical intervention remains a cornerstone in the management of POP, with various techniques employed to restore pelvic anatomy and function. Our study’s findings of significant postoperative improvements in POP-Q scores are consistent with existing literature. For instance, a study by Maher et al. (2013) demonstrated that sacrocolpopexy resulted in superior anatomical outcomes compared to vaginal approaches, with lower rates of recurrent prolapsed [16]. Laparoscopic long mesh surgery (LLMS) with augmented round ligaments is a novel uterine-preserving procedure for apical POP that has similar effective results [17]. A randomized trial by Barber et al. (2014) reported that two transvaginal surgical approaches provided effective apical support with acceptable complication rates [18]. The choice between transvaginal and abdominal approaches has been extensively debated.

Abdominal sacrocolpopexy is often considered the gold standard for apical prolapse due to its durability and lower recurrence rates. However, transvaginal approaches, including the use of synthetic mesh, have been explored to reduce operative morbidity. Notably, the FDA has issued warnings regarding the use of transvaginal mesh due to complications such as erosion and infection, which result in a decline in its utilization. A Cochrane review by Maher et al. (2016) concluded that while transvaginal mesh may reduce the risk of recurrent prolapse, it is associated with higher rates of reoperation and mesh-related complications [3].

### 4.2. Urinary Incontinence and Symptom Relief

Urinary symptoms often coexist with POP, and their resolution is a critical determinant of surgical success. Our observation of significant reductions in urinary frequency, incomplete bladder emptying, and hesitancy aligns with findings from other studies [11].

Stress urinary incontinence (SUI) is the involuntary leakage of urine due to increased abdominal pressure. Tension-free vaginal tape (TVT) and transobturator tape (TOT) are effective surgical treatments. TVT has a higher risk of bladder injury, while TOT may cause groin pain. Both procedures show comparable success rates with minimal complications [19]. The other option is the mini-sling, which is emerging as a potential gold standard for SUI treatment, offering similar efficacy and safety to traditional mid-urethral slings with reduced postoperative pain [20].

Weber et al. (2001) [21] reported that prolapse surgery led to improvements in overactive bladder symptoms in a substantial proportion of patients. Additionally, the Colpopexy and Urinary Reduction Efforts (CARE) trial found that concomitant Burch colposuspension during sacrocolpopexy reduced the incidence of postoperative SUI [21]. The study by Long et al. (2011) [20] identified that patients with preoperative urgency and higher bladder capacity were more likely to experience improvement in overactive bladder symptoms following TVM repair for POP. Our results similarly demonstrated significant reductions in urinary frequency, hesitancy, and incomplete bladder emptying after surgery [16].

The improvement in quality of life, as evidenced by decreased UDI-6 and IIQ-7 scores in our study, is corroborated by other research. Ellerkmann et al. (2001) demonstrated that surgical correction of prolapse significantly enhanced quality of life measures, including physical and social functioning [22]. However, the relationship between anatomical correction and symptom resolution is complex. A study by Brubaker et al. (2008) indicated that while anatomical success is associated with symptom improvement, some women may continue to experience urinary symptoms despite optimal surgical outcomes [23].

### 4.3. Comparison of Urodynamic Parameters

Our findings indicate that while subjective urinary symptoms improved postoperatively, objective urodynamic measures such as maximum urinary flow rate (Qmax) and maximum cystometric capacity (MCC) did not show significant changes. This discrepancy has been noted in other studies. Long CY et al. (2019) observed that urodynamic parameters may not correlate directly with symptom relief, suggesting that factors beyond measurable urodynamics contribute to patient-perceived improvement [24]. Although significant reductions in residual urine (RU) were observed, other parameters such as MCC and Qmax did not show notable changes. This discrepancy may be explained by the differing mechanisms underlying each parameter. Residual urine volume reflects incomplete detrusor contraction or outlet obstruction, which may improve following anatomical realignment and tension reduction after mesh placement. In contrast, MCC and Qmax are more influenced by intrinsic bladder compliance and detrusor contractility, which may not be directly altered by prolapse repair. Furthermore, chronic bladder outlet obstruction secondary to long-standing POP may result in detrusor underactivity that persists even after surgical correction, thus limiting improvement in these urodynamic indices. In our cohort, subjective improvement was evident despite minimal change in most urodynamic parameters, supporting the idea that subjective outcomes may not always align with objective measurements [25].

### 4.4. Clinical Implications and Future Directions

The integration of our findings with the existing literature underscores the efficacy of surgical intervention in improving both anatomical and symptomatic outcomes for women with POP. However, the variability in urodynamic findings suggests that a tailored approach, considering patient-specific factors and preferences, is essential. Future research should focus on long-term outcomes, the development of less invasive techniques with fewer complications, and a deeper understanding of the relationship between anatomical correction and functional improvement.

### 4.5. Limitations

This study has several limitations. First, the retrospective cohort design without randomization may introduce selection bias and restrict causal inference. Second, the absence of a control group limits comparison with other surgical approaches or conservative treatments. Third, while validated questionnaires (UDI-6 and IIQ-7) and urodynamic studies were used, aspects such as sexual function and patient satisfaction were not formally evaluated. Finally, this was a single-center study, and findings may not be generalizable to other populations or clinical settings.

## 5. Conclusions

The results of our study demonstrated that Calistar mesh surgery is effective in both POP anatomical restoration and associated LUTS. Although subjective urinary symptom relief is evident, we did not observe extensive significant changes in all urodynamic parameters. This may highlight the need for a comprehensive, individualized approach for POP management. Ongoing research and innovation are imperative to optimize functional outcomes and minimize complications, such as mesh exposure and sexual function following the transvaginal procedures of POP.

## Figures and Tables

**Table 1:** Baseline Characteristics of Calistar Patients (*n* = 180).

**Variable**	
Age (years)	68.60 ± 8.21
Parity (n)	3.16 ± 1.26
BMI (kg/m^2^)	24.64 ± 3.13
Pad (mL)	8.48 ± 25.27
**Past History**	
Hormone treatment	22 (12.22%)
Menopause	77 (42.78%)
Hypertension	72 (40%)
Diabetes mellitus	36 (20%)
Hysterectomy	72 (40%)
**Concomitant**	
Cervical amputation	9 (5%)
Vaginal total hysterectomy	43 (23.89%)
Urinary incontinence	85 (47.22%)

**Table 2:** Preoperative and postoperative POP-Q and quality of life scores.

Variable	Pre-Op	Post-Op	*p*-Value
Aa	1.01 ± 1.43	−1.81 ± 0.85	<0.0001
Ba	2.81 ± 1.97	−1.81 ± 0.85	<0.0001
C	0.40 ± 3.70	−6.98 ± 2.08	<0.0001
Ap	−1.77 ± 1.42	−2.27 ± 0.76	0.0033
Bp	−0.17 ± 2.43	−2.32 ± 0.58	<0.0001
Tvl	9.03 ± 1.29	8.05 ± 2.11	0.0005
UDI-6	27.78 ± 21.56	5.34 ± 10.98	<0.0001
IIQ-7	31.09 ± 30.32	2.78 ± 9.60	<0.0001

**Table 3:** Preoperative and postoperative urinary symptoms.

Symptom	Pre-Op	Post-Op	*p*-Value
Urinary frequency	45 (57.69%)	1 (1.28%)	<0.0001
Stress urinary incontinence	24 (48%)	11 (22%)	0.0033
Urge incontinence	41 (51.9%)	41 (51.9%)	<0.0001
Incomplete bladder emptying	25 (49.02%)	4 (7.84%)	<0.0001
Urinary hesitancy	55 (69.62%)	4 (5.06%)	<0.0001
Nocturia	61 (78.21%)	57 (73.08%)	0.448

**Table 4:** Urodynamic parameters before and after surgery.

Parameter	Pre-Op	Post-Op	*p*-Value
DO	74 (98.67%)	73 (97.33%)	0.3141
Qmax (mL/s)	17.19 ± 11.82	18.49 ± 8.47	0.3397
RU (mL)	132.50 ± 154.39	54.51 ± 54.22	0.0003
FS (mL)	171.93 ± 87.27	156.21 ± 86.70	0.1224
MCC (mL)	395.37 ± 139.74	372.18 ± 135.28	0.1379

## Data Availability

Data available upon request due to privacy restrictions.

## Abbreviations

The following abbreviations are used in this manuscript:

POP	Pelvic Organ Prolapse
TVM	Transvaginal Mesh
LUTS	Lower Urinary Tract Symptoms
POP-Q	Pelvic Organ Prolapse Quantification
UDI-6	Urinary Distress Inventory-6
IIQ-7	Incontinence Impact Questionnaire-7
UDS	Urodynamic Study
Qmax	Maximum Urinary Flow Rate
RU	Residual Urine
FS	First Sensation of Bladder Filling
MCC	Maximum Cystometric Capacity
Pdet	Detrusor Pressure at Maximum Flow
MUCP	Maximum Urethral Closure Pressure
FUL	Functional Urethral Length
UCA	Urethral Closure Area
DO	Detrusor Overactivity
RIG	Retractable Insertion Guide
TAS	Tissue Anchoring System
BMI	Body Mass Index
SUI	Stress Urinary Incontinence
TVT	Tension-Free Vaginal Tape
TOT	Transobturator Tape
LLMS	Laparoscopic Long Mesh Surgery
